# Thymic size is increased by infancy, but not pregnancy, nutritional supplementation in rural Gambian children: a randomized clinical trial

**DOI:** 10.1186/s12916-019-1264-2

**Published:** 2019-02-18

**Authors:** Sophie E. Moore, Anthony J. C. Fulford, Fatou Sosseh, Patrick Nshe, Momodou K. Darboe, Andrew M. Prentice

**Affiliations:** 1 MRC Unit The Gambia at the London School of Hygiene and Tropical Medicine, Atlantic Boulevard, Fajara, PO Box 273, Banjul, The Gambia; 2 Division of Women and Children’s Health, King’s College London, St Thomas’ Hospital, 10th Floor North Wing, London, SE1 7EH UK

**Keywords:** Pregnancy, Infants, Gambia, Thymus, Nutritional supplementation, DOHaD

## Abstract

**Background:**

Thymic size in early infancy predicts subsequent survival in low-income settings. The human thymus develops from early gestation, is most active in early life and is highly sensitive to malnutrition. Our objective was to test whether thymic size in infancy could be increased by maternal and/or infant nutritional supplementation.

**Methods:**

The Early Nutrition and Immune Development (ENID) Trial was a randomized 2 × 2 × 2 factorial, partially blinded trial of nutritional supplementation conducted in rural Gambia, West Africa. Pregnant women (*N* = 875) were randomized to four intervention groups (iron-folate (standard care), multiple micronutrients, protein energy or protein energy + multiple micronutrients at ‘booking’ (mean gestational age at enrolment = 13.6 weeks, range 8–20 weeks) until delivery. The iron-folate and multiple micronutrient arms were administered in tablet form and the protein energy arms as a lipid-based nutritional supplement. All intervention arms contained 60 mg iron and 400 μg folic acid per daily dose. From 24 to 52 weeks of age, infants from all groups were randomized to receive a daily lipid-based nutritional supplement, with or without additional micronutrients. Thymic size was assessed by ultrasonography at 1, 8, 24 and 52 weeks of infant age, and a volume-related thymic index calculated. Detailed data on infant growth, feeding status and morbidity were collected.

**Results:**

A total of 724 (82.7%) mother-infant pairs completed the trial to infant age 52 weeks. Thymic size in infancy was not significantly associated with maternal supplement group at any post-natal time point. Infants who received the daily LNS with additional micronutrients had a significantly larger thymic index at 52 weeks of age (equivalent to an 8.0% increase in thymic index [95% CI 2.89, 13.4], *P* = 0.002). No interaction was observed between maternal and infant supplement groups.

**Conclusions:**

A micronutrient-fortified lipid-based supplement given in the latter half of infancy increased thymic size, a key mediator of immune function. Improving the micronutrient status of infants from populations with marginal micronutrient status may improve immune development and survival.

**Trial registration:**

ISRCTN registry (controlled-trials.com) Identifier: ISRCTN49285450

**Electronic supplementary material:**

The online version of this article (10.1186/s12916-019-1264-2) contains supplementary material, which is available to authorized users.

## Background

Despite recent progress, infant and child mortality remains at unacceptably high levels in low- and middle-income countries (LMICs). Most deaths are caused by infections and it has been estimated that malnutrition contributes to 45% of all these deaths by limiting immune competence [1]. Interventions to improve nutritional status during pregnancy have been associated with better birth outcomes, including neonatal survival [2, 3] possibly through nutritionally mediated influences on infant immune development.

In early childhood, the thymus is the main site for clonal selection of lymphocytes and hence represents a key organ orchestrating the development of adaptive immunity [4]. The thymus starts to develop very early in gestation with fetal T cells detectable as early as 8 weeks. It reaches its peak size in proportion to total body weight very soon after birth and declines thereafter. Importantly, thymic size assessed in early infancy has been shown to predict subsequent survival in low-income settings [5–7].

It has long been known that the thymus is exquisitely sensitive to nutrition leading to it be described as a ‘barometer of malnutrition’ [8]. In mice, even short periods of food restriction cause profound thymic atrophy and an associated reduction in T cell numbers mediated by depressed leptin levels [9]. In humans, the thymic atrophy caused by severe acute malnutrition can be rapidly reversed by appropriate nutritional rehabilitation [10, 11] but there is no data linking thymic size and function with the milder levels of malnutrition common in LMICs.

We conducted a proof-of-concept trial in rural Gambia to test whether nutritional supplementation in early life can enhance early immune development. The ENID (Early Nutrition and Immune Development) trial was designed to combine both pregnancy and infant interventions in order to help determine the most efficacious form and timing of nutritional support. Full details of the trial protocol have been published previously [12]. We report here the primary trial outcome, the impact of intervention on infant thymic size across the first year of life.

## Subjects and methods

Full details are provided in Additional file 1: Online Supplementary Material and in the published trial protocol [12]. In brief, see the following:

### Study setting, participants and recruitment

The trial took place in the rural West Kiang region of The Gambia. All women of reproductive age (18 to 45 years) registered within the West Kiang Demographic Surveillance System [13] were invited to participate, and informed consent obtained.

Participating women were visited monthly with a short questionnaire on their last menstrual period. Height and weight were measured at the first visit and weight monthly thereafter. When a menses had been missed, a urine sample was collected for pregnancy testing using hCG kits. Women with positive results were invited for an ultrasound examination. Those confirmed as < 20 weeks pregnant by ultrasound were randomized into the trial with supplementation commencing the following week. Exclusion criteria were gestational age ≥ 20 weeks, multiple pregnancy, severe anaemia (haemoglobin (Hb) < 7 g/dL) or confirmed as HIV positive.

### Interventions: pregnancy

Women were randomized to one of four intervention arms: (1) iron-folate (FeFol) tablets, representing the usual standard of care as per Gambian Government guidelines; (2) multiple micronutrient (MMN) tablets, a combination of 15 micronutrients designed for use during pregnancy as formulated by UNICEF/WHO/UNU [14] (with the exception of iron and folate, each tablet contained 2xRDA of each micronutrient [15]); (3) protein energy and iron-folate (PE+FeFol), as a lipid-based nutritional supplement (LNS) providing the same level of iron and folate as the FeFol arm, but with the addition of energy, protein and lipids; and (4) protein energy and multiple micronutrients (PE+MMN) as the same LNS supplement fortified to provide the same level of micronutrients as the MMN arm (including FeFol). The two LNS (PE+FeFol and PE+MMN) provided an additional 746 kcal per day. The composition of the four supplements is detailed in Additional file 1: Table S1.

### Interventions: infancy

ENID trial participants were actively encouraged to exclusively breastfeed their infants by community-based health nurses. At 24 weeks of age, infants started supplementation with either an unfortified LNS paste or the same LNS formulation fortified with multiple micronutrients (Additional file 1: Table S2).

### Randomization

Randomization was performed in blocks of 8, using an automated system reflecting the 8 combinations of prenatal and infancy supplements. The antenatal arm of the trial was partly open, since it was not possible to blind project staff or study participants to the supplement type (tablet versus LNS). The infant arm of the trial was double blind, with infants receiving identically packaged formulations.

Both antenatal and infant supplements were distributed on a weekly basis by a community-based field assistant. Compliance was assessed through the collection of all unused supplements at the end of each week (tablet counts or empty/half-empty/full score for LNS).

### Procedures

At booking, 20 and 30 weeks of gestation, women were brought to the MRC Keneba clinic. At each visit, a standard antenatal examination was performed by the study midwives, with data collected on anthropometry, blood pressure, Hb and urine analysis. Fasting venous blood was collected (10 mL) and plasma and DNA stored. At the 20 and 30 week visits, fetal biometry (bi-parietal diameter, occipital frontal diameter, head circumference, abdominal circumference and femur and tibia length) was measured by ultrasound.

Where possible, project staff attended deliveries to collect and process cord blood and placenta samples [16]. Within 72 h of delivery, all women and their infants were visited by a study midwife for a general health assessment and a ‘baby check’ including infant anthropometry (weight, length, head circumference and MUAC) and gestational age assessment by Dubowitz score [17].

Infants were seen in Keneba when they were 1, 8, 12, 24 and 52 weeks of age and at home at 16, 20, 32 and 40 weeks. Thymus size was assessed sonographically at 1, 8, 24 and 52 weeks using a validated method in which the transverse diameter and the sagittal area of its largest lobe are multiplied together to give a volume-related thymic index (TI) [18]. Data are expressed as cubic centimetres but it should be noted that this is not the actual volume. Ultrasound examinations were performed by one observer (PN) to ensure consistency of measurement. Infant anthropometry was recorded at all visits. Any infant with a weight-for-length *z*-score < − 3SD from the WHO reference was considered as having severe acute malnutrition and withdrawn from the study for admission and treatment at the MRC Keneba Nutritional Rehabilitation Unit.

Data on maternal (during pregnancy only) and infant (from birth to 52 weeks) morbidity were collected on a weekly basis by questionnaire. At the weekly home visits, a questionnaire on infant feeding practices was also administered.

### Sample analyses

Hb was measured in maternal whole blood samples using a Medonic hemoglobinometer. Blood samples were separated by centrifugation and plasma frozen at − 70 °C until analysis. Plasma folate was measured by UPLC-MS/MS using an in-house assay based on a published method [19].

### Statistical analyses

Full details of the power calculations applied are provided in the published trial protocol [12]. To detect a 5% difference in infant thymic size by intervention group, with a power of 80% and at a significance level of 5%, we estimated a required total sample size of 847 mother-infant pairs. Descriptive statistics for all variables used in the analysis are presented by intervention arm. Unadjusted between-arm differences were tested using analysis of variance for continuous variables and chi-squared tests for categorical variables. Infant anthropometry was converted to *Z*-scores using the WHO Child Growth Standards [20], and SGA was defined according to a birth weight-for-gestational age < 10th percentile of the INTERGROWTH-21st standard [21]. Age of weaning was defined as the age when the infant first received weaning foods on two consecutive occasions. Infant morbidity was coded as a pooled score of the number of morbidity episodes. Episodes of diarrhoea were additionally coded as a separate variable. However, as this separation had little impact on the current analysis, we only present results using the pooled morbidity variable.

Maternal compliance with the supplement was measured by dividing the number of tablets or jars of LNS the mother consumed by the number offered, and infant compliance by dividing the number of jars given by the number of completed jars consumed, over the duration of the intervention period.

Analyses of the main outcome data were performed using either ordinary least squares regression or a random effects model fitted by generalized least squares, depending on whether each infant was represented by one or more time points. The dependent variable was the logarithm of TI. Since TI is strongly dependent on both age and size of the infant, all models included terms for infant length and its square, and, where the full age range was analysed, orthogonal polynomials up to degree four in infant age; otherwise, when a single time point was analysed, a simple linear effect for age was fitted. Terms to fit seasonality were used as previously described [22]. Results are presented according to maternal intervention only, maternal and infant intervention groups combined, and infant intervention only. Results are presented as intention to treat analyses, but analyses were also conducted using data on supplement compliance. We present data on three models: Unadjusted and only including the two main maternal intervention groups (PE and MMN); Model 1, adjusted for modifying variables selected a priori for their known association with thymic size in this environment [23] (maternal size (BMI and height), infant age, infant length, season of measurement) and (where relevant) both maternal and infant compliance; and model 2 (for analyses where infant age > 24 weeks) includes additional post-natal variables, selected as potential mediators in the association between supplement status and thymic size (infant feeding and morbidity). Effect sizes were calculated by taking the exponential of the model coefficient and its CI limits. All analyses were run in Stata 12 (StataCorp LP, College Station, Texas).

## Results

**Fig. 1:**
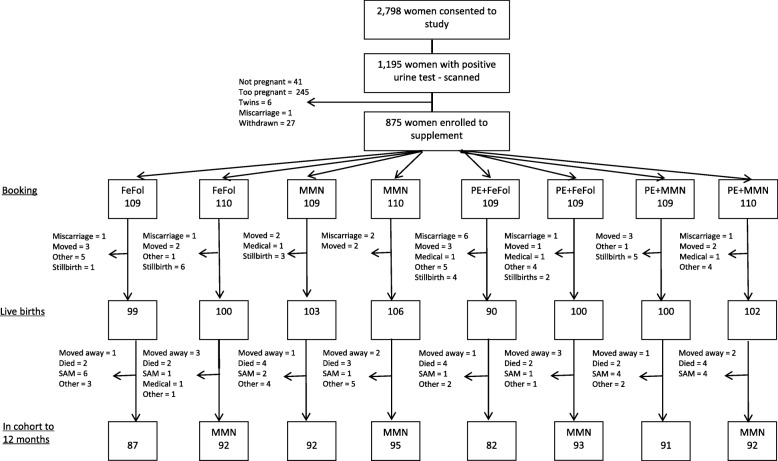
Consolidated Standards of Reporting Trials (CONSORT) diagram showing participant flow in the trial. Abbreviations: FeFol, iron-folic acid; MMN, multiple micronutrients; PE, protein energy; SAM, severe acute malnutrition. A miscarriage was defined using the WHO definition of the premature loss of a fetus up to 23 weeks of pregnancy

A total of 2798 women were enrolled for monthly surveillance. Between January 2010 and June 2013, 1195 positive hCG tests were made, followed by an ultrasound examination for pregnancy confirmation and dating. A total of 875 (73.2%) of the women scanned were eligible for enrolment and entered the antenatal supplementation phase of the trial (Fig. 1). Baseline characteristics of the women, by supplement group, are detailed in Table 1. Mean gestational age at enrolment into the trial was 13.6 weeks (range 7.0 to 20.8 weeks).

**Table 1:** Baseline characteristics of the women, by antenatal supplementation group

Maternal group	FeFol	MMN	PE+FeFol	PE+MMN
*N*	219	219	218	219
Maternal age (years)	29.3 (6.74)	30.4 (6.67)	29.0 (6.47)	29.9 (6.84)
Maternal education (years completed)^a^	1.34 (3.06)	2.03 (3.78)	1.52 (3.42)	1.31 (2.97)
Gestational age at booking (weeks)	13.8 (3.39)	13.7 (3.36)	13.6 (3.36)	13.4 (3.16)
Parity	4.30 (2.72)	3.81 (2.62)	3.95 (2.72)	4.34 (2.75)
Proportion of nulliparous women (%)	10.2	12.5	11.7	7.5
Weight (kg)	55.0 (9.0)	55.4 (9.83)	56.0 (9.29)	55.7 (10.7)
Height (cm)	161.6 (6.21)	162.1 (5.73)	161.9 (5.58)	161.6 (5.91)
BMI (kg/m^2^)	21.0 (3.23)	21.1 (3.76)	21.4 (3.30)	21.3 (3.59)
Proportion of women with BMI < 18.5 kg/m^2^	18.3	21.9	16.5	17.4
Proportion of women with BMI > 25 kg/m^2^	8.7	11.9	12.8	10.5
Haemoglobin (g/dL)	11.4 (1.40)	11.4 (1.33)	11.2 (1.39)	11.4 (1.33)
Proportion of women with haemoglobin < 11 g/dL	36.5	36.2	39.5	39.1

*Abbreviations*: *FeFol* iron-folic acid, *MMN* multiple micronutrients, *PE* protein energy, *BMI* body mass index

^a^Mean of sum of years at Koranic or English Medium school

Women were enrolled in the antenatal supplementation phase of the trial for an average of 26.1 weeks (SD = 4.4) and thus received nutritional supplements for this period. Weeks of supplementation did not differ between groups (data not shown). Women who received the tablet supplements (FeFol or MMN) had higher rates of compliance (95.7% and 93.1%, respectively) than women who received the LNS-based supplements (PE+FeFol and PE+MMN, 81.7% and 81.2%; *p* value for the difference between tablet and LNS groups < 0.0001). Measured plasma total folate levels across pregnancy support this higher supplement compliance among tablet-based supplements: Plasma total folate levels increased from baseline in both the FeFol and MMN groups, but remained at similar levels to baseline in the two LNS groups (data not presented).

A total of 75 women (8.6%) did not deliver a live born infant into the trial (Fig. 1). Of the 800 live-born infants, 660 (83%) were seen within the first 72 h for a neonatal examination including anthropometry. Mean (SD) birthweight was 3012 g (405), 9.6% of infants were born with a low birth weight (< 2500 g) and 22% of infants were born SGA. Additional file 1: Tables S3a and b detail infant birth outcomes and infant anthropometry to 6 m by antenatal supplement group and infant outcomes at 12 m by maternal and infant groups combined, and by infant groups alone. As indicated, growth faltering was endemic within this population, with weight-for-age *z*-scores (mean(SD)) declining from -0.68(0.90) to -1.15(1.04) and height-for-age from -0.56(1.03) to -0.99(1.04) over the first year of life. Exclusive breastfeeding was common in early infancy within this population, with a mean (SD) duration of exclusive breastfeeding of 5.2(1.3) months, and with 31.4% of infants exclusively breastfed to 6 months. Over the first year of life, the mean incidence of morbidity episodes (all reports summed) was 13.5 per infant, with 2.4 episodes of reported diarrhoea, 1.3 of vomiting, 4.0 of cough, 0.3 of rapid breathing, 4.9 of fever and 3.2 of ‘other’ morbidity episodes.

Mean (SD) compliance to infant supplementation was 69.5(22.6)%. Infants randomized to the MMN fortified LNS had a small but statistically significant lower mean compliance compared to infants receiving the unfortified LNS (68.3% vs 71.7%, *p* = 0.04). This did not differ by maternal supplement group.

Mean thymic index (TI) and TI to weight ratio are reported in Table 2. TI increased in absolute size from birth to 24 weeks, decreasing again by 52 weeks. However, the TI to weight ratio was largest at the measures taken in early infancy (week 1 and week 8) and smallest at 1 year of age.

**Table 2:** Thymic index (TI) and TI to weight ratio by infant age

	*N*	Thymic index (cm^3^)^a^	Thymic index to weight ratio (cm^3^/kg)
Week 1	765	9.18 (3.08)	2.78 (0.87)
Week 8	752	13.9 (4.09)	2.80 (0.76)
Week 24	747	14.7 (4.20)	2.13 (0.59)
Week 52	707	13.2 (3.71)	1.61 (0.45)

^a^Mean (±standard deviation)

**Fig. 2:**
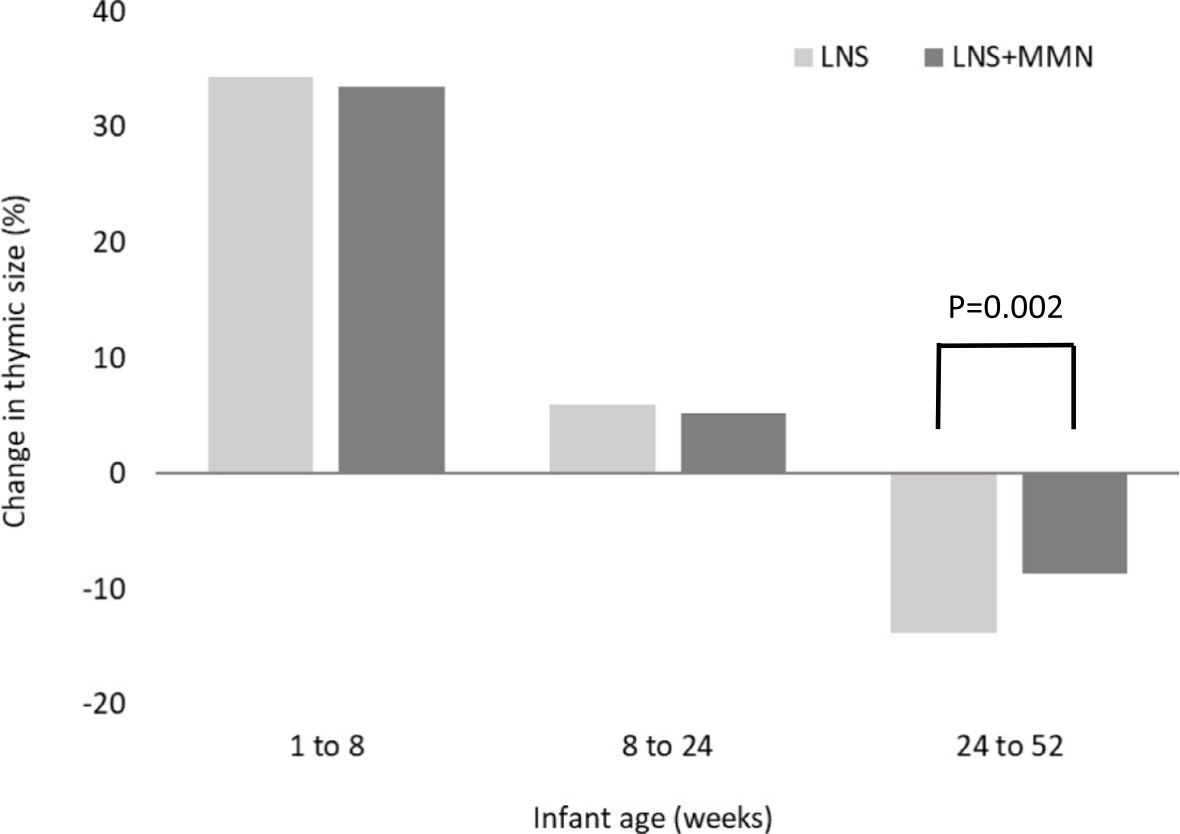
Impact of infant micronutrient supplementation on thymic index at 52 weeks of age. Percent (standard error) difference in mean thymic index between successive time points

Maternal supplementation had no consistent impact on infant TI, in either the unadjusted or adjusted models (Table 3). A borderline negative effect of supplementation with PE during pregnancy on TI at week 1 was observed, but this did not persist following adjustment for co-variables. Supplementation with MMN to infants from 24 to 52 weeks had a significant, positive impact on infant TI; infants who received the LNS fortified with MMNs had a significantly larger TI than those who received the unfortified placebo LNS (Fig. 2; + 8.04% (95% CI 2.89, 13.4), *P* = 0.002). This effect was observed when both maternal and infant groups were fitted and in both the adjusted models, with little evidence of mediation by any of the selected co-variables (Table 3). There was no significant interaction of the infant supplement with maternal supplement (data not presented). Of note, infant morbidity (sum of all morbidity episodes from 6 to 12 months of infant age) was significantly correlated with TI at 12 months of age; a large TI was associated with fewer morbidity episodes (< 0.0001; data not presented).

**Table 3:** Effect of pregnancy and infancy nutritional supplementation on infant thymic index

	Effect size (%)^a^	95% CI	*P* value
Week 1 only			
Unadjusted			
PE	− 5.03	− 9.75, − 0.05	0.05
MMN	2.16	− 2.91, 7.52	0.41
Model 1^b^			
PE	− 3.49	− 8.23,1.49	0.17
MMN	1.68	− 3.28, 6.93	0.51
All time points to infant age < 6 months			
Unadjusted			
PE	− 2.07	− 5.14, 1.12	0.20
MMN	1.71	− 1.49, 5.01	0.30
Model 1			
PE	− 0.89	− 3.85, 2.16	0.56
MMN	2.10	− 0.95, 5.24	0.18
Combined maternal and infant—week 52 only			
Unadjusted			
PE	− 2.19	− 6.84, 2.71	0.38
MMN	2.13	− 2.72, 7.25	0.39
Infant MMN	8.08	2.92, 13.48	0.002
Model 1			
PE	− 1.97	− 6.64, 2.92	0.42
MMN	2.31	− 2.56, 7.43	0.36
Infant MMN	8.50	3.28, 13.97	0.001
Model 2^c^			
PE	− 1.91	− 6.56, 2.97	0.44
MMN	2.06	− 2.79, 7.14	0.41
Infant MMN	7.99	2.81, 13.43	0.002
Infant supplement only			
Unadjusted	8.04	2.89, 13.44	0.002
Model 1	8.80	3.20, 13.81	0.001
Model 2	8.10	2.92, 13.55	0.002

*Abbreviations*: *PE* protein energy, *MMN* multiple micronutrients

^a^Effect size computed as 100 × [antilog(*β*) − 1] where *β* is the regression coefficient from regression models

^b^Model 1 adjusted for infant size, infant age, infant sex, season of measurement and maternal size (BMI and height) and (as relevant) maternal and infant compliance to supplement

^c^Model 2 (for data up to week 52 only) adjusted for the same variables as model 1, but also infant age at weaning (defined as the age of introduction of non-breast milk feeds) and infant morbidity (sum of morbidity episodes across the first year of life)

## Discussion

This randomized trial provides novel data suggesting that direct supplementation to the infant from 24 to 52 weeks of age with MMNs provided in the form of a small quantity LNS results in a modest but clear increase in thymus size. Nutritional supplementation during pregnancy conferred no benefit. These findings support previous observational data suggesting the importance of the early post-natal environment on thymus size [23, 24] and specifically support a role for supplementary micronutrients given in infancy. These findings have implications beyond thymic development; improving the micronutrient status of infants from populations with marginal status may improve immune development and survival.

The thymus is the primary lymphoid organ essential for the establishment of a normal peripheral T lymphocyte immune repertoire. However, the sonographic assessment of TI used in the current trial represents an anatomical rather than functional measure of infant immune status. Limited published data exist to correlate TI to immune function in infancy. In a small study of Danish infants (*N* = 36 measured at 10 months of age), a positive correlation was observed between TI and the proportion of CD8^+^ cells in peripheral blood and the CD4/CD8 ratio [25]. In a larger cohort study from The Gambia (*N* = 138) however, no direct association was observed between TI and lymphocyte subpopulations, although a strong seasonal influence was observed on both [26]. Notably, data from infants in Guinea-Bissau and Bangladesh indicates that a small thymus in infancy is an independent risk factor for infection-related mortality in infancy [5–7], supporting the relevance of TI as a predictor of future immunocompetence.

Existing literature suggests a beneficial effect of post-natal nutrition on thymic development. Severe PE malnutrition results in thymocyte death and thymic atrophy [27], and these effects appear to be reversible with nutritional rehabilitation [11]. A limited number of studies in animal models have investigated the impact of micronutrient deficiencies on thymic function (e.g. [28–31]), but in humans, the focus has largely been on zinc. Zinc plays a major role in cell division, differentiation, apoptosis and gene transcription and strongly influences the immune system, affecting primarily T cells [32], and zinc supplementation in severely malnourished Jamaican children was shown to increase radiographically assessed thymic size [10]. Ours is the first published trial to demonstrate a positive effect on thymic size through supplementation with multiple micronutrients. The supplement included 22 micronutrients, and so understanding the mechanism of effect is difficult. Of note, children who received the MMN fortified LNS were not significantly bigger at 52 weeks of age than those children who received the placebo supplement, and the effect on TI persisted when adjusted for infant size. Supplemented children had a non-significantly lower incidence of morbidity than the control children and children with a larger TI at 12 months of age had significantly fewer morbidity episodes during the time of supplementation from 6 to 12 months of age; however, understanding causality in this context is not possible.

We did not observe a consistent effect of any of the antenatal supplement groups on infant thymic size, as compared to the usual standard of care (daily iron and folic acid only). This result was surprising, as the supplements were purposefully designed to ensure nutritional repletion in this context. The additional micronutrients provided in the MMN and PE+MMN arms contained twice the RDA for pregnancy, with the exception of iron and folic acid which were kept consistent across all groups, in line with Gambian Government guidelines. The two LNS arms (PE+FeFol and PE+MMN) were formulated as a large quantity LNS, containing 746 kcal per daily dose. This is considerably higher than the standard 118 kcal per day used in the more typically given small quantity LNS preparations.

Our data indicate that compliance with the pregnancy LNS supplement was poor, as compared to tablets, possibly impacting the results observed. The PE supplement was administered in a daily dose, providing 746 kcal/day, with women encouraged to consume the entire dose herself, either directly from the pot or mixed with other foods. Both self-reported compliance and the available data on plasma total folate levels (data not presented here) indicate that not all women adhered to the protocol. A small qualitative study embedded within the ENID Trial focused on understanding maternal perceptions to the supplements (tablets vs LNS supplement groups) and highlighted a number of reported concerns (e.g. poor acceptability of the smell and taste of the LNS, side effects of nausea and vomiting), possibly contributing to poor compliance [DalyA, Understanding the differential effects and perceptions of lipid-based nutrient supplements compared with tablet supplements on the habitual dietary intake and behaviours of pregnant women in rural Gambia, MSc Dissertation: London School of Hygiene and Tropical Medicine, unpublished]. Supplement sharing among family members, however, was not reported. A small number of other published studies, from a variety of settings, have reported acceptability and compliance data from trials of LNS given in pregnancy and lactation. In trials using small quantity LNS products (typically 20 g, providing 118 kcal per day) in Ghana [33, 34], Malawi [34] and Bangladesh [35], no differences were observed between the LNS products and comparison groups (iron-folic acid or multiple micronutrient tablets), despite participants reporting a number of acceptability issues largely related to associated side effects (vomiting, nausea, etc.). A single study using a higher dose LNS (372 kcal/d) in pregnant women in Burkina Faso also found no statistical difference in compliance between the LNS and multiple micronutrient arms [36]. The findings of a lower compliance with the much higher dose LNS given in our trial cautions against the suitability of using such products during pregnancy.

To the best of our knowledge, this is the first published trial to look at the impact of combined maternal and infant nutritional supplementation on infant immune development, and in a population with high rates of maternal and infant undernutrition. The further strengths of the study include the individual random assignment, early detection of pregnancy complete with ultrasound dating, high retention rates, and the detailed, longitudinal assessment of the primary trial outcome, thymic index. The number of participants recruited (875 mother-infant pairs) was consistent with the a priori sample size calculation [12], providing sufficient power to test the primary hypothesis of effect by both maternal and infant supplement group. The primary limitation was the poorer compliance to the antenatal LNS arms of the trial and low compliance with the infant supplement. Supervised supplementation may have avoided this issue.

## Conclusion

In conclusion, we have shown that infant thymic size is increased in rural Gambian infants supplemented with small quantity LNS containing multiple micronutrients. No effect was observed with either PE, MMN or combined PE and MMN supplementation when given to the mother during pregnancy, and there was no apparent interaction between antenatal and infant administered MMN supplementation. Because the effect on TI was independent of an effect on infant size, we conclude a direct benefit on infant thymic size of MMN administered during infancy. The immunomodulatory consequences of this increase in thymus size, and the longevity of the effect, require further investigation.

## Additional file

Additional file 1: Online supplementary material. Table S1: Nutritional composition of the allocated daily intake of pregnancy supplements; Table S2: Nutritional composition of the allocated daily dose (20 g) of the infant supplements; Table S3a: Infant outcomes at birth at 24 weeks of age, by antenatal intervention arms; Table S3b Infant outcomes at 52 weeks of age, by infant intervention arms. Microsoft Word file. (DOCX 53 kb)
