# Supplementary material for: Thymic size is increased by infancy, but not pregnancy, nutritional supplementation in rural Gambian children: a randomized clinical trial
Source: BMC Med. 2019 Feb 18;17:38. doi: 10.1186/s12916-019-1264-2 (PMC6378709; doi:10.1186/s12916-019-1264-2)
Supplement: Supplementary file 1 — Online supplementary material. Table S1: Nutritional composition of the allocated daily intake of pregnancy supplements; Table S2: Nutritional composition of the allocated daily dose (20 g) of the infant supplements; Table S3a: Infant outcomes at birth at 24 weeks of age, by antenatal intervention arms; Table S3b Infant outcomes at 52 weeks of age, by infant intervention arms. Microsoft Word file. (DOCX 53 kb) [file 12916_2019_1264_MOESM1_ESM.docx]

Thymic development is improved by infancy, but not pregnancy, nutritional supplementation in rural Gambian children: A randomized clinical trial.

**Supplementary online material**

**Supplementary methods**

**Study setting, participants and recruitment**

The trial took place from April 2010 to February 2015 in the West Kiang region of The Gambia, a rural subsistence farming community of savannah and farmland, approximately 160 km from the capital Banjul, and with a total resident population of approximately 15,000. Following approval from the local community, all women of reproductive age (18 to 45 years), resident in any one of the 36 villages in West Kiang and registered within the West Kiang Demographic Surveillance System (DSS) ^1^ were invited to participate, and informed consent obtained. The trial was approved by the joint Gambia Government / MRC Unit The Gambia Ethics Committee (Project number SCC1126v2).

Each month from enrolment, participating women were visited by a member of the study team with a short questionnaire on the date of their last menstrual period (LMP). Height and weight were measured at the first visit, and weight at each monthly visit thereafter. When a menses had been missed, a urine sample was collected and transported to the laboratory at MRC Keneba for pregnancy testing using hCG kits (QuickVue™ One-Step hCG Urine Test, bioMérieux, UK). Women with a positive test were then invited to the clinic at MRC Keneba for an ultrasound examination. Those confirmed as <20 weeks pregnant by ultrasound (using a Siemens ACUSON Antares Ultrasound Imaging System (Siemens Medical Solutions USA, Inc., California, USA with a CH6-2 (5.71 MHz) transducer), were randomized into the trial, with supplementation commencing the following week. Women were excluded from the trial on the basis of: gestational age ≥20 weeks; multiple pregnancy; severe anaemia (haemoglobin < 7 g/dL); or confirmed as HIV positive, following voluntary counselling and testing (VCT) by an MRC Keneba midwife (see below).

**Interventions: Pregnancy**

On confirmation of pregnancy by ultrasound, women were randomized to one of four intervention arms: 1.) Iron-folate (FeFol), representing the usual standard of care during pregnancy, as per Gambian Government guidelines; 2.) Multiple micronutrients (MMN), a combination of 15 micronutrients, specifically designed for use during pregnancy as formulated by UNICEF/WHO/UNU ^2^. With the exception of iron and folate, each tablet contained 2 X RDA of each micronutrient ^3^. Both the FeFol and MMN supplements were formulated as tablets and manufactured by Scanpharm, Birkerød, Denmark. 3.) Protein-energy and iron-folate (PE+FeFol), A lipid based nutritional supplement (LNS) providing the same level of iron and folate as the FeFol arm, but with the addition of energy, protein and lipids. 4.) Protein-energy and multiple micronutrients (PE+MMN). A micronutrient fortified LNS supplement providing the same level of micronutrients to the MMN arm (including FeFol), in addition to the energy and protein and lipid content. The two LNS products were manufactured by Valid Nutrition, Nairobi, Kenya. The composition of the four pre-natal supplement groups is detailed in **Table 1**.

**Interventions: Infancy**

In The Gambia, exclusive breastfeeding to six months of age is recommended and actively promoted. Between birth and six months post-partum, women in the ENID Trial were encouraged to exclusively breastfeed their infants and supported by community-based health nurses. At six months of age infants started supplementation with either an unfortified LNS paste, or the same LNS formulation fortified with multiple micronutrients (**Table 2**). The infant supplements were manufactured by Valid Nutrition, Nairobi, Kenya .

**Randomization**

Randomization was performed in blocks of 8, using an automated system with the 8 groups reflecting the 8 combinations of prenatal and infancy supplements. Thus, once randomised, the code allocated to each mother was the same for her and her infant. Allocation of each supplement combination to a number between 1 and 8 was performed by a person independent to the trial, and this information was passed directly to the supplement manufacturers. Each box of intervention product was then labelled by supplement code only. The antenatal arm of the trial was partly-open, since it was not possible to blind project staff or study participants to the supplement type (tablet versus LNS). However, within types, supplements were packaged in individually labelled plastic pots, with identical packaging. The infant arm of the trial was double blind, with infants receiving identically packaged formulations prepared in individually labelled plastic containers.

Antenatal supplements were given to pregnant women on a weekly basis by a field assistant resident in the community. Compliance was assessed through the collection of all unused supplements at the end of each week. For tablets, a count on remaining tablets was performed and for LNS products a ‘score’ based on the amount of supplement left remaining in the jar was made (empty, half-empty, full). In addition, random spot checks were performed on 10% of the women/infants each week and used/unused supplement details recorded. Infant supplements were distributed to mothers/carers on a weekly basis, with the mother/carer instructed to administer the product on a daily basis by mixing approximately 20 g with the infant’s normal weaning food. Identical spoons were provided to mothers/carers, to encourage that the specified amount was given each day. Compliance was assessed in the same way as for the antenatal supplements.

**Procedures**

At booking, 20 and 30 weeks gestation, women attended the clinic at MRC Keneba for a detailed antenatal assessment. At each visit, a standard antenatal examination was performed by the study midwives, with data collected on blood pressure, haemoglobin (Hb) and urine analysis. At the booking visit women were also offered Voluntary Couselling and Testing (VCT) for HIV, as is routine practice in West Kiang. Data were additionally collected on maternal anthropometry (weight, standing height, sitting height, lower leg length, waist and hip circumference and mid-upper-arm circumference) all using standard, regularly validated equipment and following standard operating procedures. A fasting 10 mL sample of venous blood was collected, and plasma and extracted DNA stored for future analysis. At the 20 and 30 weeks gestation visit, fetal biometry data were also collected by ultrasound (Siemens ACUSON, as above) including bi-parietal diameter, occipital frontal diameter, head circumference, abdominal circumference and femur and tibia length.

Where possible, deliveries were attended by project staff, and cord blood and placenta samples collected and processed ^4^. Within 72 hours of delivery, all women and their newborn infants were visited by a study midwife for a general health assessment and a ‘baby check’, including infant anthropometry (weight, length, head circumference and MUAC) and gestational age assessment by Dubowitz score ^5^.

Following delivery, infants were then seen at MRC Keneba when they were 1, 8, 12, 24 and 52 weeks of age, and at home at 16, 20, 32 and 40 weeks of age. Full details of these study visits are given in the published trial protocol ^6^. Thymus size was assessed sonographically at 1, 8, 24 and 52 weeks of age using a validated method in which the transverse diameter of the thymus and the saggital area of its largest lobe are multiplied together to give a volume-related thymic index (TI) ^7^. TI was measured using a Siemens Acuson ultrasound unit (as before) together with a P10-4 Transducer (Siemens, Camberley, UK). Ultrasound examinations were performed by one observer (PN) to ensure consistency of measurement. At each visit where TI was measured, detailed measurements of infant anthropometry were taken (weight, length, knee-heel length, head circumference, abdominal circumference, chest circumference, thigh circumference, MUAC, and triceps, biceps, subscapular, suprailiac and thigh skinfold thickness), using standard protocols with regularly validated equipment.

Data on maternal (during pregnancy only) and infant (from birth to 52 weeks of age) morbidity were collected on a weekly basis by questionnaire. For women, incidence and frequency (number of days, 1-7) of fever, diarrhoea, nausea/vomiting, dizziness, dysuria, vaginal itching/discharge, abdominal pain, oedema, bleeding and ‘other’ (details specified) was recoded at these weekly visits. For infants, incidence and frequency of diarrhoea (> 2 loose stools/day), vomiting (not associated with feeding), cough, rapid breathing, fever and ‘other’ (with details specified) were recorded. At these weekly visits, a questionnaire on infant feeding practices was also administered, with details collected on breast feeding status (are you currently breastfeeding your infant?) and non-breast milk feeds (collection of semi-quantitative information on non-breast milk feeds given in the proceeding 7 days).

**Sample analyses**

Hb was measured in maternal whole blood samples in the MRC Keneba laboratory using a haemoglobinometer (Medonic CA 530 Haematology Analyser, Boule Medical AB, Stockholm, Sweden). Blood samples were then separated by centrifugation and plasma was frozen at −70 °C until analysis. Plasma folate was measured at MRC Human Nutrition Research, Cambridge, UK by UPLC-MS/MS and using an in house assay based on a published method ^8^. Briefly, this method uses a methanol protein crash followed by solid phase extraction on phenyl columns to separate folates from other analytes in the plasma. Stable isotope labelled internal standards are added during the extraction step and undergo processing identical to the analytes thereby normalizing for sample preparation and instrument variability. Highly specific detection of the analytes and internal standards is accomplished by UPLC-MS/MS analysis and the ratio of analyte to internal standard signal is compared to that of a calibration curve to determine analyte concentration.

**Statistical analyses**

Descriptive statistics for all variables used in the analysis are presented by intervention arms. Unadjusted between-arm differences were tested using analysis of variance for continuous variables and Chi-squared tests for categorical variables. Infant anthropometry was converted to Z-scores using the WHO Child Growth Standards ^9^. Age of weaning was defined as the age when the infant first was reported to receive weaning foods on two consecutive weeks. This was fitted either as a simple linear effect, regardless of the time point (since the weaning age probably reflects mother and/or infant characteristics both prior to and after weaning as much as it does the infant’s intake at a particular time point) or as binary variable indicating whether the infant was exclusively breastfeed at each time point. Infant morbidity was coded as a pooled score of the number of morbidity episodes. Episodes of diarrhoea were additionally coded as a separate variable.

Maternal supplement compliance data was coded as 0 (full), 0.5 (half full) or 1 (empty). This was then used to calculate compliance by dividing the number of jars of LNS the mother consumed by the number she was offered, over the duration of the intervention period. For the two tablet arms, compliance was calculated by dividing the number of tablets consumed by the total number offered.

Women randomised to receive the LNS based supplements were given 1 jar per day, in a one week supply of 7 jars and women randomised to tablets received a weekly supply of 14 tablets (2 per day). Infant compliance was measured by dividing the number of jars of supplement given (one jar per week) by the number of completed pots of supplement consumed, over the duration of the intervention period.

Analyses of the main outcome data were performed using either ordinary least squares regression or a random effects model fitted by generalized least squares, depending on whether each infant was represented by one or more time points. The dependent variable was the logarithm of TI. Since TI is strongly dependent on both age and size of the infant all models included terms for infant length and its square, and, where the full age range was analysed, orthogonal polynomials up to degree four in infant age; otherwise, when a single time point was analysed, a simple linear effect for age was fitted. Terms to fit seasonality were used as previously described ^10^. Data are presented according to maternal intervention only, and maternal and infant intervention groups combined. Data are presented as intention to treat analyses and as models including compliance data. We present data on three models: Unadjusted, and only including the two maternal intervention groups (PE and MMN); Model 1, adjusted for *a priori* selected modifying variables (maternal size (BMI and height), infant age, infant length, season of measurement); and Model 2 includes additional post-natal exposure variables (infant feeding and morbidity). Effect sizes were calculated by taking the exponential of the model coefficient and its CI limits. All analyses was run in Stata 12 (Statacorp LP, College Station, Texas).

**References**

1. Hennig BJ, Unger SA, Dondeh BL, et al. Cohort Profile: The Kiang West Longitudinal Population Study (KWLPS)-a platform for integrated research and health care provision in rural Gambia. *International journal of epidemiology.* 2015.

2. UNICEF/WHO/UNU. *Composition of a multi-micronutrient supplement to be used in pilot programs among pregnant women in developing countries.* New York: UNICEF;1999.

3. Kaestel P, Michaelsen KF, Aaby P, Friis H. Effects of prenatal multimicronutrient supplements on birth weight and perinatal mortality: a randomised, controlled trial in Guinea-Bissau. *European journal of clinical nutrition.* 2005;59(9):1081-1089.

4. Jobarteh ML, Moore SE, Kennedy C, Gambling L, McArdle HJ. The effect of delay in collection and processing on RNA integrity in human placenta: experiences from rural Africa. *Placenta.* 2014;35(1):72-74.

5. Dubowitz LM, Dubowitz V, Palmer P, Verghote M. A new approach to the neurological assessment of the preterm and full-term newborn infant. *Brain & development.* 1980;2(1):3-14.

6. Moore SE, Fulford AJ, Darboe MK, Jobarteh ML, Jarjou LM, Prentice AM. A randomized trial to investigate the effects of pre-natal and infant nutritional supplementation on infant immune development in rural Gambia: the ENID trial: Early Nutrition and Immune Development. *BMC pregnancy and childbirth.* 2012;12:107.

7. Hasselbalch H, Nielsen MB, Jeppesen D, Pedersen JF, Karkov J. Sonographic measurement of the thymus in infants. *European radiology.* 1996;6(5):700-703.

8. Fazili Z, Whitehead RD, Jr., Paladugula N, Pfeiffer CM. A high-throughput LC-MS/MS method suitable for population biomonitoring measures five serum folate vitamers and one oxidation product. *Analytical and bioanalytical chemistry.* 2013;405(13):4549-4560.

9. Group WMGRS. *WHO Child Growth Standards: methods and development: length/height-for-age, weight-for-age, weight-for-length, weight-for-height, and body mass index-for-age. .* Geneva, Switzerland: WHO;2006.

10. Fulford AJ, Rayco-Solon P, Prentice AM. Statistical modelling of the seasonality of preterm delivery and intrauterine growth restriction in rural Gambia. *Paediatric and perinatal epidemiology.* 2006;20(3):251-259.

**Supplementary Tables**

**Table S1: Nutritional composition of the allocated daily intake of pregnancy supplements**

|  | **Tablets** | | **LNS** | |
| --- | --- | --- | --- | --- |
|  | **FeFol** | **MMN** | **PE+FeFol** | **PE+MMN** |
| Iron (mg) | 60 | 60 | 60 | 60 |
| Folate (μg) | 400 | 400 | 400 | 400 |
| Vitamin A (RE μg) |  | 1600 | 2.85 | 1600 |
| Vitamin D (IU) |  | 400 | - | 400 |
| Vitamin E (mg) |  | 20 | 4.2 | 20 |
| Vitamin C (mg) |  | 140 | 2.25 | 140 |
| Vitamin B1 (mg) |  | 2.8 | 0.3 | 2.8 |
| Vitamin B2 (mg) |  | 2.8 | 0.45 | 2.8 |
| Niacin (mg) |  | 36 | 1.35 | 36 |
| Vitamin B6 (mg) |  | 2.8 | 0.15 | 2.8 |
| Vitamin B12 (μg) |  | 5.2 | 0.1 | 5.2 |
| Zinc (mg) |  | 30 | 3.3 | 30 |
| Copper (mg) |  | 4 | 1.05 | 4 |
| Selenium (μg) |  | 130 | 6.15 | 130 |
| Iodine (μg) |  | 300 | 2.6 | 300 |
| Energy (kcal) |  |  | 746 | 746 |
| Protein (g) |  |  | 20.8 | 20.8 |
| Lipids (g) |  |  | 52.6 | 52.6 |

Abbreviations: FeFol, iron folic acid; MMN, multiple micronutrients; PE, protein energy; LNS, lipid based nutritional supplement.
Data in shaded cells represent MMN content from base ingredients

**Table S2: Nutritional composition of the allocated daily dose (20g) of the infant supplements**

| **Nutrient** | **LNS** | **LNS+MMN** |
| --- | --- | --- |
| β-Carotene (μg RE) | 1.84 | 400 |
| Vitamin C (mg) | 1.88 | 30 |
| Folic acid (μg) | 13.1 | 80 |
| Thiamine (mg) | 0.06 | 0.3 |
| Riboflavin (mg) | 0.04 | 0.4 |
| Vitamin B3 (mg) | 0.32 | 4 |
| Pantothenic acid (mg) | 0.08 | 1.8 |
| Vitamin B6 (mg) | 0.02 | 0.3 |
| Vitamin B12 (μg) | 0.06 | 0.5 |
| Vitamin D (μg) | 0.34 | 5 |
| Vitamin E (mg) | 0.12 | 2.7 |
| Vitamin K (μg) | 1.40 | 10 |
| Iron (mg) | 0.46 | 9 |
| Zinc (mg) | 0.24 | 4 |
| Calcium (mg) | 33.1 | 100 |
| Potassium (mg) | 91.76 | 152 |
| Copper (mg) | 0.02 | 0.2 |
| Selenium (μg) | 1.44 | 10 |
| Iodine (μg) | 1.40 | 90 |
| Phosphorus (mg) | 42.56 | 82 |
| Magnesium (mg) | 14.56 | 16 |
| Manganese (mg) | 0.08 | 0.08 |
| Total energy (kcal) | 108 | 108 |
| Linoleic acid (g) | 1.29 | 1.29 |
| Linolenic acid (g) | 0.29 | 0.29 |

Abbreviations: LNS, lipid-based nutritional supplement; MMN, multiple micronutrients.
Data in shaded cells represent MMN content from base ingredients

**Table S3a: Infant outcomes at birth at 24 weeks of age, by antenatal intervention arms.**

| Maternal Group | **FeFol** ^a^ | **MMN** | **PE** | **PE+MMN** | ***P-*value** |
| --- | --- | --- | --- | --- | --- |
| **Birth** | | | | | |
| WAZ | -0.65 (0.86) | -0.62 (0.93) | -0.61 (0.88) | -0.60 (0.85) | 0.94 |
| LAZ | -0.08 (0.97) | -0.10 (1.05) | -0.13 (1.01) | 0.01 (0.97) | 0.62 |
| SGA (%) | 21.7 | 23.4 | 22.6 | 23.0 | 0.95 |
| **6 months** | | | | | |
| WAZ | -0.71 (1.14) | -0.69 (1.08) | -0.71 (1.09) | -0.77 (1.20) | 0.92 |
| LAZ | -0.47 (1.00) | -0.43 (1.09) | -0.47 (1.02) | -0.56 (1.11) | 0.67 |
| WLZ | -0.48 (1.27) | -0.48 (1.16) | -0.48 (1.10) | -0.49 (1.27) | 0.99 |
| Haemoglobin (g/dL) | 10.48 (1.61) | 10.44 (1.39) | 10.43 (0.999) | 10.65 (1.05) | 0.43 |
| Morbidity ^b^ | 4.55 (3.28) | 4.11 (3.35) | 4.20 (3.23) | 4.36 (3.24) | 0.55 |

Abbreviations: FeFol, Iron-Folic Acid; MMN, Multiple micronutrients; PE, Protein-energy; WAZ, Weight-for-age z-score; LAZ, Length-for-age z-score; WLZ, Weight-for-length z-score; SGA, Small-for-gestational age.

^a^ Mean (± Standard Deviation)

^b^ Mean number of morbidity episodes from birth to 24 weeks of age. .

**Table S3b Infant outcomes at 52 weeks of age, by infant intervention arms.**

| Infant Group | LNS | LNS+MMN | *P*-value |
| --- | --- | --- | --- |
| WAZ | -1.22 (1.05) | -1.09 (1.04) | 0.18 |
| LAZ | -1.05 (0.98) | -0.945 (1.09) | 0.20 |
| WLZ | -0.96 (1.07) | -0.85 (1.04) | 0.16 |
| Haemoglobin (g/dL) | 9.8 (1.61) | 9.9 (1.62) | 0.34 |
| Morbidity* | 5.5 (3.19) | 5.2 (3.21) | 0.16 |
|  |  |  |  |

Abbreviations: LNS, Lipid-based nutritional supplement; MMN, Multiple micronutrients; WAZ, Weight-for-age z-score; LAZ, Length-for-age z-score; WLZ, Weight-for-length z-score.

^a^ Mean (± Standard Deviation)

^b^ Mean number of morbidity episodes from 24 to 52 weeks of age. .
